# Identification and expression analysis of genes associated with bovine blastocyst formation

**DOI:** 10.1186/1471-213X-7-64

**Published:** 2007-06-08

**Authors:** Karen Goossens, Ann Van Soom, Mario Van Poucke, Leen Vandaele, Jo Vandesompele, Alex Van Zeveren, Luc J Peelman

**Affiliations:** 1 Department of Nutrition, Genetics and Ethology, Faculty of Veterinary Medicine, Ghent University, Heidestraat 19, B-9820 Merelbeke, Belgium; 2 Department of Reproduction, Obstetrics and Herd Health, Faculty of Veterinary Medicine, Ghent University, Salisburylaan 133, B-9820 Merelbeke, Belgium; 3 Centre for Medical Genetics Ghent, Ghent University Hospital, Medical Research Building, De Pintelaan 185, B-9000 Ghent, Belgium

## Abstract

**Background:**

Normal preimplantation embryo development encompasses a series of events including first cleavage division, activation of the embryonic genome, compaction and blastocyst formation.

First lineage differentiation starts at the blastocyst stage with the formation of the trophectoderm and the inner cell mass. The main objective of this study was the detection, identification and expression analysis of genes associated with blastocyst formation in order to help us better understand this process. This information could lead to improvements of *in vitro *embryo production procedures.

**Results:**

A subtractive cDNA library was constructed enriched for transcripts preferentially expressed at the blastocyst stage compared to the 2-cell and 8-cell stage. Sequence information was obtained for 65 randomly selected clones. The RNA expression levels of 12 candidate genes were determined throughout 3 stages of preimplantation embryo development (2-cell, 8-cell and blastocyst) and compared with the RNA expression levels of *in vivo *"golden standard" embryos using real-time PCR. The RNA expression profiles of 9 (75%) transcripts (*KRT18*, *FN1*, *MYL6*, *ATP1B3*, *FTH1*, *HINT1*, *SLC25A5*, *ATP6V0B*, *RPL10*) were in agreement with the subtractive cDNA cloning approach, whereas for the remaining 3 (25%) (*ACTN1*, *COPE*, *EEF1A1*) the RNA expression level was equal or even higher at the earlier developmental stages compared to the blastocyst stage. Moreover, significant differences in RNA expression levels were observed between *in vitro *and *in vivo *produced embryos. By immunofluorescent labelling, the protein expression of KRT18, FN1 and MYL6 was determined throughout bovine preimplantation embryo development and showed the same pattern as the RNA expression analyses.

**Conclusion:**

By subtractive cDNA cloning, candidate genes involved in blastocyst formation were identified. For several candidate genes, important differences in gene expression were observed between *in vivo *and *in vitro *produced embryos, reflecting the influence of the *in vitro *culture system on the embryonic gene expression. Both RNA and protein expression analysis demonstrated that *KRT18*, *FN1 *and *MYL6 *are differentially expressed during preimplantation embryo development and those genes can be considered as markers for bovine blastocyst formation.

## Background

The low success rate of *in vitro *embryo production procedures in cattle, together with early embryonic mortality, lead to the loss of a large number of potential calves, retarded genetic progress and consequently the loss of money and time for the cattle breeding industry. Despite improvements on the culture media and culture conditions only 30–40% of the fertilised oocytes reach the blastocyst stage and it is generally accepted that the quality and developmental competence of *in vitro *produced (IVP) bovine embryos has failed to keep up with those of their *in vivo *counterparts [1].

Normal preimplantation embryo development in cattle is characterised by several cleavage divisions of the fertilised egg, activation of the embryonic genome around the 8–16 cell stage, compaction and blastocoel formation leading to the blastocyst. Before the major activation of the embryonic genome, the bovine preimplantation embryo is controlled by maternal genomic information that is accumulated during oogenesis [2]. Both the maternal and embryonic gene expression occurs in a stage- and time-dependent manner [3].

The first important differentiation events take place at the blastocyst stage, resulting in the generation of two distinct cell lineages: the trophectoderm cells (TE) and the inner cell mass (ICM). The ICM gives rise to the embryo, whereas the TE forms the placenta. Since these two lineages are divergent in morphological and biochemical aspects, it is reasonable to hypothesise that many differentiation-related genes are expressed at this stage. Genes being expressed in the blastocyst stage but not in earlier stages are therefore functional candidates for the regulative processes that take place at the onset of differentiation [4]. The identification of novel genes involved in blastocyst formation and the analysis of their expression patterns may help us to understand the mechanisms that control blastocyst formation and may help us to understand why certain embryos do not make it through this stage. As *in vitro *culture conditions do not fully mimic the *in vivo *situation and as it has been shown that morphological and physiological differences [5-7] as well as differences in gene expression [8-11] exist between *in vivo *and *in vitro *cultured embryos, the RNA expression levels of *in vitro *produced embryos should be compared with those of *in vivo *"golden standard" embryos. Changes in transcript abundance between *in vitro *and *in vivo *embryos may help to assess the normality of *in vitro *produced embryos and may help to optimise the *in vitro *culture conditions.

In previous studies, genes involved in preimplantation embryo development were identified using techniques such as differential display reverse transcription PCR [4,12,13], large scale cDNA library construction [14-16], cDNA microarray [17-19] and suppression subtractive hybridisation (SSH) [4,20-22]. Subtractive cDNA cloning [23] is a powerful tool for the detection of stage-specific transcribed genes. In contrast to e.g. microarray analysis, this PCR-based method can be accomplished without prior knowledge of the genes being expressed, and yields subtracted cDNA pools that are differentially expressed. However, further confirmation of the outcome is required by means of real-time PCR, which is a perfect technique not only to validate the subtractive cDNA cloning results, but also to gather quantitative data concerning the selected transcripts. In the present study subtractive cDNA cloning was used to detect genes that are differentially expressed in the bovine blastocyst compared to genes present in 2-cell and 8-cell stage embryos. The differential RNA expression status of 12 subtracted cDNA clones was validated throughout 3 stages of preimplantation embryo development (2-cell, 8-cell and blastocyst) and compared with the RNA expression levels of their *in vivo *"golden standard" counterparts using real-time PCR. By immunofluorescent labelling, the protein expression of 3 genes with high differences in RNA expression levels between the developmental stages was examined during preimplantation embryo development to check whether the protein expression patterns were comparable with the RNA expression levels.

## Results

### Construction and screening of the SSH library

A subtractive cDNA library was constructed for the enrichment of transcripts preferentially expressed in *in vitro *cultured blastocysts relative to 2-cell and 8-cell embryos.

As bovine embryos contain limited amounts of mRNA and as the subtractive cDNA cloning requires at least 0.5 μg of starting material, a linear amplification step was performed using the SMART PCR cDNA synthesis Kit. By using 27 PCR cycles for linear amplification, the formation of non-specific PCR products due to overcycling was prevented.

The amplification products enriched for blastocyst specific genes were cloned, single clones were randomly picked and single-pass sequenced. Partial sequence information was obtained for 65 clones. Those partial sequences were compared with known sequences in the Genbank by doing a BLAST analysis against the mammal nr database. BLAST searches revealed that 54 sequences were homologous to 36 different known genes, 5 sequences were homologous to genomic sequences and 2 clones had no matches in the database and could not be identified. The 4 remaining clones were false positives, giving similarity with the cloning vector. Among the identified genes, we found 14 genes coding for ribosomal proteins and 10 mitochondrial genes.

The sequence data of the clones were submitted to the NCBI dbEST database [24] and the accession numbers and their putative identities are listed in Table 1.

**Table 1:** Overview of the blastocyst specific subtracted cDNA clones compared to known sequences in Genbank

**EST Accession Number**	**Gene name**	**Function**	**Accession Number BlastN**	**% Identity**
DQ347577	*B. taurus *γ-actin Cytoplasmic 2	Cytoskeletal protein	NM_001033618	99% 443/447
DQ347578				100% 237/237
DQ347579	*B. taurus *actinin, α 1	Cytoskeletal protein	XM_591685	99% 257/258
DQ347580	*B. taurus *Fibronectin 1	Cytoskeletal protein	XM874396	95% 457/478
DQ347581	*H. sapiens *Microtubule-actin crosslink factor 1	Cytoskeletal protein	NM_012090	88% 425/478
DQ347582				89% 317/354
DQ347583	*B. taurus *Myosin light chain prot. 6	Cytoskeletal protein	BTMYO217	99% 413/417
DQ347584	*B. taurus *cytokeratin 18	Cytoskeletal protein	XM_582930	99% 442/443
DQ347585				96% 219/228
DQ347586	*B. taurus *Adenin nucleotide translocator 2	Transporter activity	AB065433	97% 272/279
DQ347587				97% 245/259
DQ347588				99% 300/301
DQ347589				98% 297/303
DQ347590	*B. taurus *Ferritin heavy chain polypeptide 1	Iron storage	NM_174062	98% 171/174
DQ347591				99% 230/231
DQ347592			AF540563	100% 167/167
DQ347593				100% 170/170
DQ347594				95% 356/372
DQ347595	*B. taurus *Histidine triad nucleotide binding protein 1	Hydrolase	NM_175812	95% 350/366
DQ347596				95% 340/356
DQ347597	*B. taurus *coatomer protein complex, subunit ε	retrograde Golgi-to-ER transport	X76980	99% 364/366
DQ347598	*B. taurus *neural precursor cell expressed, developmental down-regulated 8	linkage of ubiquitin	AF227256	99% 173/174
DQ347599	*H. sapiens *tyrosine 3-monooxygenase	signal transduction	BC010352	91% 77/84
DQ347600	*H. sapiens *ATPase Na/K transporting, subunit b3	Osmoregulation	NM_001035393	99% 212/214
DQ347601				98% 175/178
DQ347634	*B. taurus *cyclin G-associated kinase	Cell cycle	NM_001046084	95% 226/236
DQ347637				96% 130/135
DQ347603	*B. taurus *elongation factor 1 α	Translation	AJ238405	89% 305/341
DQ347604	*B. taurus *ribosomal prot. L3	Translation	NM_174715	100% 167/167
DQ347605	*B. taurus *ribosomal prot. L6		NM_001031756	99% 133/134
DQ347606	*H. sapiens *ribosomal prot. L7		NM_001014928	99% 423/426
DQ347607	*B. taurus *ribosomal prot. L10/QM		NM_174760	99% 166/167
DQ347608	*B. taurus *ribosomal prot. L17		AB099021	96% 256/265
DQ347609	*B. taurus *ribosomal prot. L23		NM_001035014	99% 330/331
DQ347610	*B. taurus *ribosomal prot. S5		NM_001015531	98% 465/470
DQ347611	*H. sapiens *ribosomal prot. S11		NM_001024568	99% 289/290
DQ347613	*B. taurus *ribosomal prot S8		NM_001025317	99% 333/334
DQ347612	*B. taurus *ribosomal prot. S12		BC102500	98% 386/390
DQ347614	*B. taurus *ribosomal prot. 40S S26-2-like		NM_001015561	98% 331/336
DQ347615	*B. taurus *ribosomal prot. S27		XM_880953	99% 287/289
DQ347616	*B. taurus *ribosomal prot. large P2		NM_174788	98% 443/452
DQ347617	*B. taurus *U1 snRNP-specific prot.		XM_872762	99% 331/333
DQ347602	*B. taurus *ATPase H+ transporter V0 subunit b	Energy metabolism	NM_001038038	100% 634/634
DQ347636	*B. taurus *cytochrome C oxidase 7A2	Energy metabolism	NM_175807	94%199/210
DQ347618	*B. taurus *mitoch. genome ATP6	Energy metabolism	AY526085	100% 251/251
DQ347619	*B. taurus *mitoch. genome ND4			99% 250/251
DQ347620	*B. taurus *mitoch. genome COX3			98% 374/381
DQ347621	*B. taurus *mitoch. genome COX2			99% 225/226
DQ347622	*B. taurus *mitoch. genome COX1			97% 260/268
DQ347623	*B. taurus *mitoch. genome COX3			98% 259/264
DQ347624	*B. taurus *mitoch. genome COX2			99% 219/220
DQ347625	*B. taurus *mitoch. genome COX2			99% 226/227
DQ347626	*B. taurus *mitoch. genome COX1			100% 240/240
DQ347627	*B. taurus *mitoch. genome COX2			99% 429/430
DQ347629	*B. taurus *BAC library clone rp42-147e22	Unknown	AC092858	98% 400/406
DQ347631				95% 141/147
DQ347628	*B. taurus *BAC library clone rp42-518P7	Unknown	AC129959	94% 218/230
DQ347630				98% 193/195
DQ347632	*B. taurus *BAC library clone CH240-472P12	Unknown	AC150855	90% 142/157
DQ347633	Unknown EST	Unknown	No similarity found	
DQ347635				

### Quantification of specific transcripts by real-time PCR

Real-time RT-PCR analysis was performed for 12 genes in order to make a relative quantification of the RNA levels in the 3 studied bovine developmental stages and to check for differences in RNA levels between *in vivo *and *in vitro *produced embryos.

Primers were designed for a selection of 12 identified genes, based on their gene function. The primer information (gene symbol, gene name, amplicon accession number, GenBank accession number and % identities with Genbank sequences) is listed in Table 2. Gene-specific amplification was confirmed for the 12 primer pairs by sequencing, by a single peak in melt-curve analysis and a single band with the expected size in agarose gel-electrophoresis. No primer-dimer formation was detected and the standard curves derived from 10-fold serial dilutions of pooled cDNA gave correlation coefficients greater than 0.99 and efficiencies between 82 and 105%. The RNA expression levels of the selected genes were measured in duplicate in 3 to 6 single *in vitro *and *in vivo *produced embryos from the 3 studied stages of preimplantation development (2-cell, 8-cell and blastocyst).

**Table 2:** Details of the primers used for PCR analysis

Gene Symbol	Gene Name	Amplicon Acc. Number	Genbank Acc. Number	% Identity
ACTN1	Actinin, alpha 1	DQ347561	XM_603102	100%
ATP1B3	Na/K ATPase, beta 3 subunit	DQ347562	EST SSH	100%
ATP6V0B	H+ ATPase, V0 subunit B	DQ347563	XM_582011	100%
COPE	coatomer protein complex, subunit epsilon	DQ347564	NM_176673	100%
EEF1A1	Elongation factor 1 alpha	DQ347565	AJ238405	100%
FN1	Fibronectin 1	DQ347566	K00800	100%
FTH1	Ferritin heavy chain polypeptide 1	DQ347567	NM_174062	99%
HINT1	Histidine triad nucleotide binding protein 1	DQ347568	NM_175812	98%
KRT18	Cytokeratin 18	DQ347569	XM_582930	100%
MYL6	Myosin light chain protein 6	DQ347572	NM_175780	100%
RPL10	Ribosomal protein L10/QM	DQ347575	NM_174760	100%
SLC25A5	Adenin nucleotide translocator 2	DQ347576	NM_174659	100%

To correct for any variation in both mRNA content and differences in enzymatic efficiencies, the quantitative results were normalised to the geometric mean of the 3 best reference genes as described in our previous study [25]. The normalised values for each target transcript were analysed using the 95% confidence intervals.

**Figure 1:**
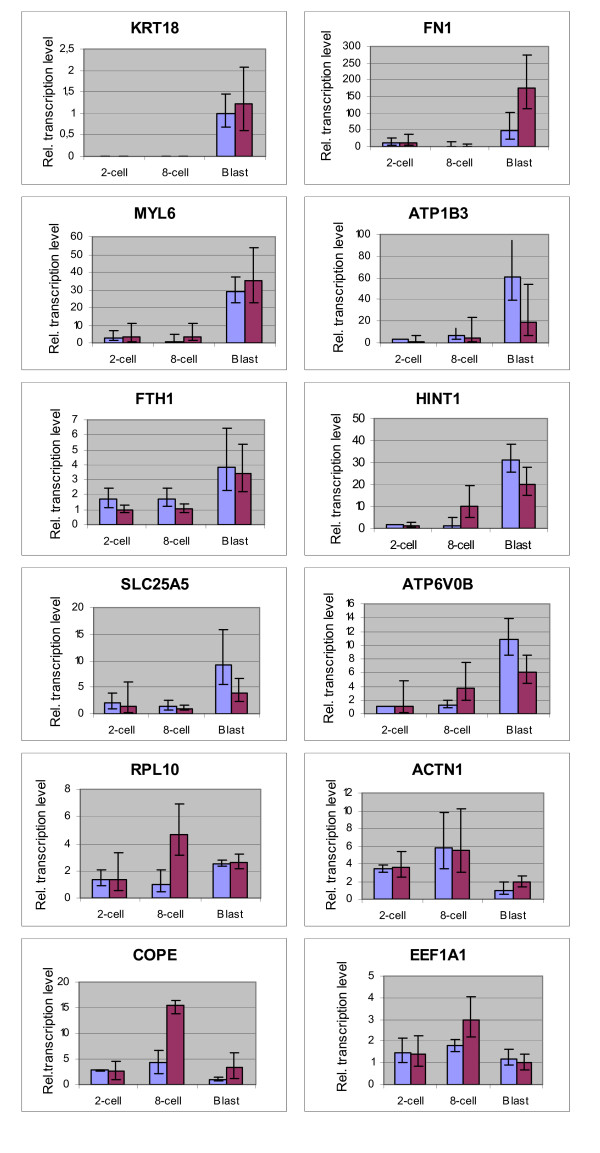
**Comparison of the relative expression levels of 12 genes at 3 stages of bovine embryo development**. Mean relative abundance of 12 transcripts at 3 different developmental stages (2-cell, 8-cell and blastocyst) of *in vitro *(lilac-coloured bars)and *in vivo *(claret-coloured bars) produced bovine embryos, determined by real-time qPCR.

The mean relative abundances of the gene transcripts studied are shown in Figure 1.

*KRT18 *was not detected up to the blastocyst stage, and no differences were observed between *in vitro *and *in vivo *produced embryos. For *MYL6 *and *FN1 *the RNA levels were significantly higher at the blastocyst stage compared to the earlier stages. Remarkable is the 3.5-fold higher *FN1 *RNA expression in *in vivo *produced blastocysts compared with their *in vitro *produced counterparts.

The differences between the blastocysts and the 2/8-cell stage embryos for *FTH1 *were smaller but significant in both *in vitro *and *in vivo *produced embryos.

*ATP1B3, HINT1, SLC25A5, ATP6V0B *and *RPL10 *had significantly lower RNA levels in *in vitro *produced 2/8-cell embryos compared to the *in vitro *produced blastocysts, but surprisingly no significant differences exist between the *in vivo *produced 8-cell and *in vivo *produced blastocyst embryos. This is due to a lower RNA level in the *in vivo *produced blastocysts (*ATP1B3 *and *SLC25A5*) on the one side and a higher RNA level at the 8-cell stage (*RPL10*) on the other side or a combination of both (*HINT1 *and *ATP6V0B*).

In contrast, 3 genes (*ACTN1*, *COPE *and *EEF1A1*) showed a relative expression pattern that was not in agreement with the results obtained by SSH. The RNA levels for those 3 genes were highest at the 8-cell stage and decreased significantly at the blastocyst stage. Especially for *COPE *but also for *EEF1A1 *the *in vivo *produced 8-cell embryos had a significantly higher RNA level than their *in vitro *produced counterparts.

### Immunofluorescent labelling

**Figure 2:**
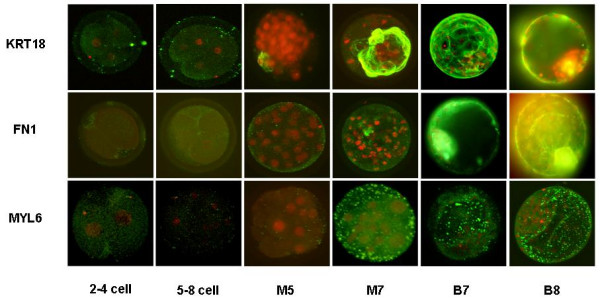
**Results of the immunofluorescent experiments for KRT18, FN1 and MYL6**. Confocal laser scanning images of *in vitro *produced bovine embryos labelled with primary mouse antibodies for KRT18, FN1 and MYL6 respectively in combination with FITC-labelled secondary goat-anti-mouse antibodies. The nuclei are stained with propidium iodide. Different stages of preimplantation embryo development were analysed (2–4 cell, 5–8 cell, M5: morula day 5 p.i., M7: morula day 7 p.i., B7: blastocyst day 7 p.i., B8: blastocyst day 8 p.i.).

Immunofluorescent labelling was performed for KRT18, FN1 and MYL6 on *in vitro *produced embryos of different developmental stages (2–4 cell, 5–8 cell, morula day 5 p.i., morula day 7 p.i., blastocyst day 7 p.i. and hatched blastocyst day 8 p.i) to verify whether the protein expression showed the same pattern as the RNA expression. The results of the immunofluorescent labelling experiments are shown in Figure 2.

**Figure 3:**
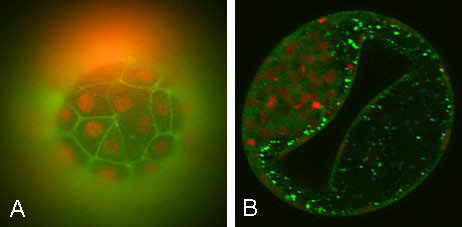
**KRT18 and MYL6 expression in *in vitro *produced bovine blastocysts**. A) Image of the surface of a bovine blastocyst day 8 p.i.labelled for KRT18. The nuclei were stained with propidium iodide. KRT18 was expressed at the cell-cell contact sites of the trophectoderm cells. B) Image of a midplane section through a bovine blastocyst day 8 p.i. labelled with antibodies for MYL6. The nuclei were stained with propidium iodide. MYL6 proteins were located in the trophectoderm cells surrounding the blastocoel cavity.

No KRT18 expression was measured in the 2–4 cell and 5–8 cell embryos except for a few small positive spots on the surface of the embryos, which may be caused by remainders of cumulus cells. The first onset of the KRT18 protein expression was detected at the morula day 5 p.i. and at the morula day 7 p.i. a ring of KRT18 positive cells was observed. At the blastocyst stage, KRT18 was predominantly expressed in the TE cells, whereas little or no expression was measured in the ICM. As shown in Figure 3A, KRT18 was detected at the cell-cell contact sites of the TE cells.

FN1 was not detected in the first developmental stages. The first onset of FN1 expression was seen in 7-day old morulae and it was clearly present at the blastocyst stages. But in contrast to KRT18, FN1 was very distinct in the ICM, forming filamentous structures between the TE and the ICM.

No MYL6 protein expression was seen before 7 days p.i MYL6 was strongly expressed at the late morula, the blastocyst and hatched blastocyst stage. In Figure 3B one can appreciate that MYL6 is located in the TE cells surrounding the blastocoel cavity.

The images of the positive and negative control experiments are supplemented in additional file 1.

The results of the immunofluorescent experiments were consistent with the RNA expression patterns during preimplantation embryo development.

## Discussion

In the present study a subtractive cDNA library was constructed between blastocyst embryos as the tester population and a pool of 2-cell and 8-cell embryos as the driver population, in order to enrich for genes differentially expressed at the blastocyst stage.

Sequence information was obtained for 65 randomly picked clones. Out of these 65 ESTs representing the blastocyst embryos, 10 were homologous to mitochondrial genes and 14 to ribosomal genes. Mitochondria play an essential role in many events during early development. In cattle, the mtDNA copy number increases during blastocyst expansion and hatching, consistent with an increase in mitochondrial RNAs. This indicates that the transcriptional activation of the mitochondrial genome coincides with the pronounced structural and functional differentiation of the mitochondria during preimplantation development [26]. The largest group of ESTs was identified as genes involved in protein synthesis and included several ribosomal proteins. The high degree of ribosomal protein mRNAs corresponds with previous data showing that between the 2-cell stage and the blastocyst stage, the level of ribosomal protein mRNA is said to increase about 20-fold [27]. The protein content of *in vivo *derived preattachment cattle embryos increases 2-fold from the 2-cell through to the blastocyst stage [28]. This rise in protein content is correlated with rapid cell divisions at the late morula and early blastocyst stage [29,30].

Real-time qPCR was used to verify the SSH results and at the same time to compare the gene expression between *in vitro *produced embryos and *in vivo *golden standard embryos. The geometric mean of *GAPD*, *YWHAZ *and *SDHA *was used for normalisation. Vandesompele and colleagues [31] demonstrated that the conventional use of a single gene for normalisation leads to relative large errors and validated the geometric mean of multiple carefully selected housekeeping genes as an accurate normalisation factor. In Goossens and colleagues [25] it was demonstrated that *GAPD*, *YWHAZ *and *SDHA *were stably expressed reference genes, suitable for normalisation of quantitative data within different preimplantation embryo stages. The problem of measuring RNA expression levels throughout preimplantation development is confounded by the fact that cell numbers and cell sizes are constantly changing during this developmental interval. To allow ontogenic analysis, the embryos were compared as a single unit and the reference genes will correct for the differences between the embryos. Other normalisation strategies, like normalisation against total cell number, normalisation against RNA mass quantity or the use of exogenous control RNA (spike) [32,33] are not assumption free. Spikes correct for differences in RNA extraction and differences in enzymatic efficiencies but do not account for the quality and quantity of input sample.

In most studies about gene expression in embryos, only the RNA expression was considered, but the differences in RNA expression do not always imply differences on protein level, as there may be regulation at the translation level as well. By immunofluorescent labelling using mouse antibodies, the protein expression of KRT18, FN1 and MYL6 was evaluated for the first time during bovine preimplantation embryo development. Furthermore, the location of the protein expression in the embryo can tell us more about the specific function of the protein during the process of blastocyst formation.

Both *KRT18 *mRNA and protein expression were found to be absent at the 2-cell and 8-cell stage and abundant at relatively high levels at the blastocyst stage, demonstrating the embryonic origin of this transcript. KRT18 was only expressed in the cell-cell contact sites of the TE, but not in those of the ICM. This makes that *KRT18 *can be seen as a marker for differentiation, adding to previous described markers for trophoblast differentiation in mice as there are *Cdx2 *and *Eomes *[34-36]. As TE is a kind of specialized epithelium, genes involved in de novo differentiation of an epithelium, including gene families encoding for cell polarity, cell junctions, cytoskelet formation and ion transport [37,38] are involved in TE differentiation. *KRT18 *is a cytoskeletal protein and is found, together with *KRT8*, in simple epithelia where they heterodimerise to form the intermediate filaments. They influence the 3-D formation of cell-cell or cell-substrate contacts in embryonic visceral endoderm [39-41]. Stanton and Green [42] reported in mouse embryos a progressive rise for both KRTs from the 2-cell to the blastocyst stage, paralleling the development of the TE. Targeted deletion of *KRT18 *in the mouse leads to trophoblast fragility and early embryonic lethality [43]. Other cytoskeletal proteins such as α-catenin, β-catenin, occludin, zonula occludens protein-1 and connexin43 are also expressed at the cell-cell contact sides of the TE cells [44], suggesting a co-localisation with KRT18.

FN1 was found to be significantly higher expressed in blastocysts compared to the earlier stages, and this difference was even 3.5× higher in the *in vivo *produced embryos compared to the *in vitro *produced ones. FN1 protein was predominantly expressed in the ICM and formed filamentous structures between the TE and the ICM. The differential expression of *FN1 *in blastocyst stage embryos of several mammalian species, including bovine, has previously been reported by other authors [4,22,45-47] and this gene has been associated with blastocyst formation. It is an adhesive extracellular matrix component and performs a vital role during cell proliferation, cell adhesion and cell mobility. It exists as a homodimer and is composed of several domains such as a heparin binding domain, a fibrin binding domain, a collagen binding domain and a cell recognition domain. This provides FN1 with the opportunity to interact with and bind to several ligands such as cells, heparin, fibrin, collagen, immunoglobulins and DNA [48]. Because events such as cell proliferation, cell adhesion and cell mobility are essential during early embryogenesis, FN1 does qualify as an important participant during this phase of embryo development. Besides, Aplin and colleagues [46] reported that FN1 acts as a bridging ligand between the collagen matrix and integrins at the cytotrophoblast surface, mediating anchorage and/or migratory activity in the process of implantation. The vital importance of FN1 for normal embryo development has been demonstrated convincingly since mouse embryos lacking this protein die shortly after gastrulation from mesodermal defects [49]. The significant difference in *FN1 *mRNA expression levels which we noticed between *in vivo *and *in vitro *produced bovine embryos has also been reported by Mohan and colleagues [10]. Lower RNA levels exhibited by *in vitro *produced embryos may be responsible for the poor quality of these embryos compared to their *in vivo *counterparts.

Myosin light chains associate with the motor protein myosin and are believed to play a role in the regulation of its actin-based ATPase activity. RNA levels of *MYL6*, the smooth muscle isoform of myosin light chain, were significantly higher at the blastocyst stage compared to the earlier stages. No significant differences were seen between *in vivo *and *in vitro *produced embryos. MYL6 is involved in the cytoskeletal organisation [50] which is in agreement with the observed protein expression in the TE cells surrounding the blastocoel cavity. The expression of MYL6 proteins around the blastocoel may also contribute to the statement of Niimura [51] who inferred that contractions in mouse blastocysts occur by activation of myosin light chain kinase resulting in the phosphorylation of myosin light chains and causing the efflux of blastocoelic fluid.

*ATP1B3 *shows a significant difference in RNA levels between the 2/8-cell stage and the blastocyst stage of *in vitro *produced embryos, but remarkably this difference is negligible for *in vivo *produced embryos. The Na/K ATPase consists of a catalytic α subunit and a noncatalytic, glycosylated β subunit, each encoded by multigene families [52]. The β subunit is required for structural and functional maturation of the α subunit [53]. Blastocyst formation involves the establishment of a transtrophectoderm ion gradient mediated by the Na/K ATPase which pumps water through water channels called aquaporins [28]. Accumulation of fluid in the blastocoel is essential for differentiation of the ICM and TE cell types [54]. The gene expression patterns of several Na/K ATPase subunits have been reported previously in mouse [55] and bovine [56], and Adjaye and colleagues [57] identified *ATP1B3 *as a marker specific for the TE in human blastocysts using a cDNA microarray but as far as we know, this is the first study in which the RNA expression levels of *ATP1B3 *in bovine embryos are described. The higher *ATP1B3 *RNA levels in *in vitro *produced blastocysts might be an explanation for the faster blastulation of *in vitro *produced embryos compared to *in vivo *produced ones. Van Soom and colleagues [58] have demonstrated that *in vivo *morulae display a more firm and prolonged compaction and that they start blastulation at a later embryonic age and cell number, moreover the addition of serum to the culture medium seemed to enhance blastocyst development [59]. *In vivo *morulae develop more gradually to the blastocyst stage and this might go together with a slower rise in *ATP1B3 *expression. Another explanation for the higher *ATP1B3 *expression *in vitro *might be a compensation for the lower expression of another NA/K ATPase subunit or another functional analogue, but this was not further investigated.

The differences between the blastocysts and the 2- and 8-cell stage embryos for *FTH1 *were smaller but significant and no differences were observed between *in vivo *and *in vitro *produced embryos. FTH1 has an important role in the control of intracellular iron distribution and the constitution of long term iron stores. The ferroxidase activity associated with the H subunit is necessary for iron uptake by the ferritin molecule. *FTH1*-/- mice embryos die between 3.5 and 9.5 days of development [60] demonstrating that the ferritin H subunit is indispensable for embryonic development. *FTH1 *was also present in the human blastocyst SSH library as reported by Morozov *et al*. [61].

The mRNA levels for *ATP6V0B*, *HINT1*, *RPL10 *and *SLC25A5 *were higher at the blastocyst stage compared to the earlier stages for *in vitro *produced embryos, but no significant differences existed between the *in vivo *produced 8-cell and *in vivo *produced blastocyst embryos. The qPCR results of those genes for *in vitro *produced embryos confirmed the results of the subtractive cDNA library.

The RNA expression patterns of the remaining genes (*ACTN1*, *COPE *and *EEF1A1*) were not in agreement with the subtractive cDNA cloning results. Those genes were abundant at an equal amount or higher at the earlier stages. Those false positives prove the requirement for the verification of the subtractive cDNA outcome and are in agreement with the reported 30% false positives obtained by subtractive cDNA cloning in previous studies [10]. The significantly higher RNA levels *in vivo *produced 8-cell embryos for *COPE *but also for *EEF1A1 *is an important observation. *COPE *is a subunit of the coatomer protein complex and is involved in the retrograde Golgi-to-ER transport of dilysine-tagged proteins, whereas *EEF1A1 *is responsible for the enzymatic delivery of aminoacyl tRNAs to the ribosome. Both genes are involved in protein synthesis. Around the 8–16 cell stage the embryonic genome becomes active, resulting in the synthesis of embryonic proteins. The lower expression of genes involved in protein synthesis (*COPE*, *EEF1A1 *and *RPL10*) in *in vitro *8-cell embryos might indicate that the embryonic protein synthesis is affected or delayed in *in vitro *embryos.

Differences in gene expression between *in vivo *and *in vitro *produced embryos reflect the effects of the *in vitro *culture system on the transcriptional activity [11]. Preimplantation embryos are capable of developing in a wide range of culture conditions. However, this adaptation of the embryo to suboptimal conditions may result in a lower embryo quality. It will be a challenge for the future to determine the importance of the affected genes for the process of embryo development and to change the culture conditions *in vitro *in order to induce the expression of those genes to levels identical to those under *in vivo *conditions, and in this way improve the quality of the embryo.

## Conclusion

The results of this study add to the knowledge of the process of bovine blastocyst formation. Several genes were identified as candidate markers involved in blastocyst formation. The fact that some of those candidate genes were differentially expressed between *in vivo *and *in vitro *produced embryos confirms the belief that culture conditions influence embryonic gene expression. Further functional analyses of these candidate genes may help to optimise *in vitro *embryo culture systems in order to improve the quality of *in vitro *produced bovine embryo.

## Methods

### *In vitro *embryo production

Bovine embryos were produced *in vitro *as described by Yuan and colleagues [62]. Briefly, bovine oocytes were aspirated from ovaries collected at a local slaughterhouse. Immature cumulus-oocyte complexes were selected from follicular fluid, washed three times in HEPES-TALP and matured for 22 to 26 hr in groups of 100 in 500 μl maturation medium at 39°C in a humidified 5% CO_2 _incubator. After maturation, the oocytes were inseminated with frozen-thawed sperm from one bull (1 × 10^6 ^spermatozoa/ml). After 20 hr the cumulus cells and spermatozoa were mechanically removed from the presumptive zygotes, which were placed in groups of 25 in 50 μl droplets of synthetic oviduct fluid supplemented with 5% fetal calf serum and cultured up to the desired stages at 39°C in 5% CO_2_, 5% O_2 _and 90% N_2_. The embryos were collected at the indicative time period after fertilisation: 2-cell (24–28 hr p.i.), 8-cell (48–52 hr p.i.), blastocyst (day 8). All embryos were washed three times in PBS, pooled and frozen at -80°C until RNA extraction.

### *In vivo *embryo production

The *in vivo *embryo production protocol has been approved by the local ethical committee and was done after superovulation and artificial insemination of 4 Holstein cows for the collection of 2-cell and 8-cell embryos and 2 Holstein heifers for the collection of blastocysts. Oestrus cycles of the donor cows and heifers were synchronized by two intramuscular injections (on day 1 and on day 12) with 526 mg of cloprostenol (Estrumate^® ^– Shering-Plough, Belgium), followed by oestrus detection 2 days later. Donor cows were intramuscularly injected with 8 equal doses of Stimufol^® ^(Université de Liège, Belgium) (500 μg porcine FSH-100 μg porcine LH) over 4 days, where as heifers received a lower total dosis of Stimufol^® ^(Université de Liège, Belgium) (250 μg porcine FSH-50 μg porcine LH) in 8 decreasing doses over 4 days. Stimufol administration was started at day 10 in the cows and at day 11 and 12 in the heifers in order to group the embryo collections. To induce luteolysis, 789 mg cloprostenol (Estrumate^® ^– Shering-Plough, Belgium) was injected along with the 5^th ^injection of pFSH/pLH. All donors were checked for oestrus 48 h after the cloprostenol injection and artificially (AI) inseminated twice with two straws of semen of a bull of established fertility on 12 and 24 hr after the onset of the oestrus. The same bull was used for in vivo and in vitro insemination. The 2-cell and 8-cell embryos were recovered 36 hours respectively 84 hours after AI. After collection of the reproductive tract at the slaughterhouse, the fallopian tube and the uterus were both flushed with approximately 250 ml PBS supplemented with 2% FCS. Blastocysts were recovered by non-surgical uterine flushing on day 7 and 8 after AI. After epidural anaesthesia with 5 ml Procaine HCL 4%^® ^(procainehydrochloride, VMD, Belgium), each horn was flushed with 0.5 l of PBS.

All *in vivo *embryos were washed four times in Hepes-Talp and subsequently three times in PBS before freezing at -80°C.

### Construction of the SSH library

#### mRNA extraction, cDNA synthesis and amplification

Poly(A)+ RNA was isolated from a pool of 40 IVP blastocysts (day 8) as a tester population and a pool of 40 IVP embryos at the 2-cell and 40 at the 8-cell stage as a driver population, using the Oligotex Direct Mini Kit (Qiagen, the Netherlands) according to the manufacturer's instructions.

Due to the small amounts of mRNA extracted from bovine embryos, both tester and driver mRNA were reverse transcribed and amplified using the SMART PCR cDNA synthesis Kit (Takara Bio Inc., France). This kit is optimised for the amplification of cDNA without altering the original expression ratios [63,64] and is widely used in combination with the subtractive hybridisation procedure to study gene expression during embryo development [4,10,30,65,66].

#### Suppression Subtractive Hybridisation

The SSH was performed with the PCR-Select cDNA Subtraction Kit (Takara Bio Inc., France) following the manufacturer's instructions. Amplified double-stranded cDNA from the tester and driver was *Rsa*I digested, and 2 different adaptors were ligated to 2 fractions of the tester population. A first hybridisation was performed with an excess of driver cDNA, followed by a second hybridisation between the mixed tester fractions in the presence of an excess driver. After the second hybridisation, tester cDNA was subjected to two rounds of PCR to enrich the tester specific cDNA fragments. The amplified products were cloned into the pCR 2.1 vector (Invitrogen, Belgium) and transformed into competent DH5α *E. coli *cells (Invitrogen, Belgium).

Clones were randomly picked, the DNA-inserts were sequenced (Thermo Sequenase Primer Cycle Sequencing Kit, Amersham Bioscience, the Netherlands) with the ALF Express sequencer (Amersham Bioscience, the Netherlands) and identified using the BLAST algorithm [67].

### Quantification of specific transcripts by real-time PCR

#### RNA extraction and cDNA amplification

For the quantification of the mRNA expression levels of 12 genes obtained by SSH, total RNA was isolated from 3 to 6 *in vivo *and *in vitro *produced single embryos per developmental stage (2-cell, 8-cell and blastocyst) using the PicoPure RNA Isolation Kit (Arcturus, USA) according to the manufacturer's instructions. An in solution DNase digestion followed by an RT minus control was performed as described by Goossens and colleagues [25]. The first-strand cDNA synthesis and linear amplification were done using the WT-Ovation RNA Amplification system (NuGEN, The Netherlands) as described in the manufacturer's instructions. After the RT reaction and the linear amplification step, the cDNA was 50 times diluted in 10 mM Tris HCl pH 8.0.

#### Primer design

Sequence-specific primers were designed for 12 identified genes by the Primer 3 software [68] (Table 2). The specificity of the primers was tested using a BLAST analysis against the genomic NCBI database. PCR amplicons were characterised using Mfold [69] in order to account for any secondary structures which might influence the PCR efficiency. The PCR products were cloned (pCR 2.1 vector, Invitrogen, Belgium) and sequenced for verification (Thermo Sequenase Primer Cycle Sequencing Kit, Amersham Bioscience, the Netherlands) with a ALF Express sequencer (Amersham Bioscience, the Netherlands).

#### Real-time PCR reactions

All PCR reactions were performed in a 15 μl reaction volume on the iCycler iQ Real-Time PCR Detection System (Bio-Rad, Belgium) using the Platinum SYBR Green qPCR SuperMix-UDG (Invitrogen, Belgium) and 200 nM of each specific primer. The PCR program consisted of an initial UDG incubation step at 50°C for 2 minutes and an initial denaturation step at 95°C for 3 minutes to activate the *Taq *DNA polymerase, followed by 45 cycles of denaturation at 95°C for 20 seconds and a combined primer annealing/extension at the specific annealing temperature for 40 seconds during which fluorescence was measured. A melt curve was produced to confirm a single gene-specific peak and to detect primer-dimer formation by heating the samples from 70 to 95°C in 0.5°C increments with a dwell time at each temperature of 10 seconds while continuously monitoring the fluorescence. PCR efficiencies were calculated using a relative standard curve derived from a pooled cDNA mixture (a 10-fold dilution series with five measuring points). Each reaction was run in duplicate in the same run, whereby a no-template control was included.

The geometric mean of three reference genes, *YWHAZ*, *GAPD *and *SDHA*, was used to calculate an accurate normalisation factor as described by Goossens and colleagues [25]. The mean quantity of each transcript (raw data) was divided by the respective normalisation factor to obtain a normalised value for each transcript. The sample with the lowest value was assigned the value 1. The normalised target values were divided by the calibrator normalised target values to generate the relative expression levels.

The normalised gene expression levels were analysed by calculating 95% confidence intervals after logarithmic transformations, whereby non-overlapping intervals denote significant differences at the 0.05 level.

### Immunofluorescent labelling

Pools of 10 embryos per developmental stage (2–4 cell (24–28 hr p.i.), 5–8 cell (48–52 hr p.i.), morula day 5 p.i., morula day 7 p.i., blastocyst day 7 p.i. and hatched blastocyst day 8 p.i) were selected from the culture media, washed 3 times in PBS and fixed with 4% paraformaldehyde (Sigma, Belgium) in PBS for 1 hour at 4°C.

After washing in polyvinyl pyrrolidone (PVP; 1 mg PVP/ml PBS), the embryos were permeabilized with 0.5% Triton X-100 (Sigma, Belgium) in PBS for 30 min at room temperature. Non-specific binding sites were blocked with 10% goat serum in PVP for 30 min. After 3 washes in PVP the embryos were incubated with the mouse primary antibodies against FN1, KRT18 and MYL6 respectively (Table 3) for 2 hours at 37°C, washed again 3 times in PVP and then incubated with FITC-labeled goat-anti-mouse secondary antibodies (Molecular Probes, Invitrogen, Belgium) for 1 hour at 37°C in the dark.

**Table 3:** Primary antibodies used for immunofluorescent experiments

Antigen	Primary antibody	Isotype	Supplier	Dilution	Secondary antibody	Supplier	Dilution
KRT18	Anti KRT18 C-04	Mouse IgG1	Abcam	1/100	Goat-anti- mouse FITC	Molecular probes	1/100
FN1	Anti FN1 CSI 005–17	Mouse IgG1	Abcam	1/100	Goat-anti- mouse FITC	Molecular probes	1/100
MYL6	Anti MYL6 A01	Mouse polyclonal	Abnova	1/50	Goat-anti- mouse FITC	Molecular probes	1/100

The nuclei were stained with 0.5% propidium iodide (PI; Molecular Probes, Invitrogen, Belgium) for 30 min at room temperature. After 2 washes in PVP the embryos were mounted in a drop of glycerol with 1,4-diazabicyclo (2.2.2) octane (25 mg/ml) on slides with vaseline bridges. Negative (by replacing the primary antibody with goat serum) and double negative controls (only PI staining) were performed simultaneously to check for non-specific binding of the secondary antibody and for auto-fluorescence. A monolayer of cultured cumulus cells was used as a positive control. The results of the control experiments were supplemented in additional file 1.

Samples were examined by fluorescence microscopy (Leica DMR, Van Hopplynus, Belgium) and by confocal laser scanning microscopy (Leica TCS SP2 laser scanning spectral confocal system linked to a Leica DM IRB inverted microscope, Leica Microsystems GmbH, Germany). A krypton-argon ion laser was used for the simultaneous excitation of fluorescence for proteins and PI for DNA. All the labelling experiments were performed in duplicate.

## Authors' contributions

KG performed all the experimental procedures and was the primary author of the manuscript. AVS contributed to the design of the IVF experiments. MVP participated in the study design and provided real-time support. LV helped with the production of the in vivo embryos and the evaluation of the immunofluorescent experiments. JV provided expert input in data analysis and statistics. AVZ and LJP participated in the design of the project, helped to draft the manuscript and supervised the study. All authors read and approved the final manuscript.

## Supplementary Material

Additional File 1 **Negative and positive controls for immunofluorescent labelling experiments**. Negative (by replacing the primary antibody with goat serum) and double negative controls (only PI staining) were performed to check for non-specific binding of the secondary antibody and for auto-fluorescence. A monolayer of cultured cumulus cells was used as a positive control. (A: negative control for KRT18, B: double negative control for KRT18, C: positive control for KRT18; A': negative control for FN1, B': double negative control for FN1, C': positive control for FN1; A": negative control for MYL6, B": double negative control for MYL6, C": positive control for MYL6). Click here for file
