# Supplementary figures and images for: Identification and expression analysis of genes associated with bovine blastocyst formation
Source: BMC Dev Biol. 2007 Jun 8;7:64. doi: 10.1186/1471-213X-7-64 (PMC1899496; doi:10.1186/1471-213X-7-64)

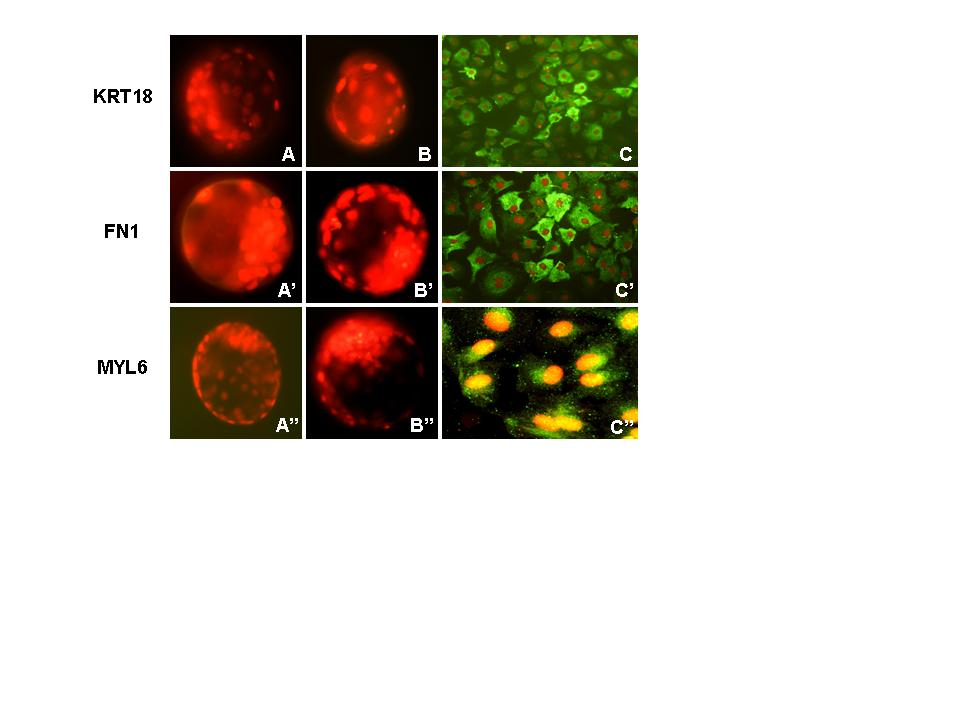

Supplement: Additional File 1 — Negative and positive controls for immunofluorescent labelling experiments. Negative (by replacing the primary antibody with goat serum) and double negative controls (only PI staining) were performed to check for non-specific binding of the secondary antibody and for auto-fluorescence. A monolayer of cultured cumulus cells was used as a positive control. (A: negative control for KRT18, B: double negative control for KRT18, C: positive control for KRT18; A': negative control for FN1, B': double negative control for FN1, C': positive control for FN1; A": negative control for MYL6, B": double negative control for MYL6, C": positive control for MYL6). [file 1471-213X-7-64-S1.jpeg]
